# Leveraging technology-driven strategies to untangle omics big data: circumventing roadblocks in clinical facets of oral cancer

**DOI:** 10.3389/fonc.2023.1183766

**Published:** 2024-01-03

**Authors:** Kshreeraja S. Satish, Kamatchi Sundara Saravanan, Dominic Augustine, Ganesan Rajalekshmi Saraswathy, Sowmya S. V, Samar Saeed Khan, Vanishri C. H, Shreshtha Chakraborty, Prizvan Lawrence Dsouza, Kavya H. N, Ibrahim F. Halawani, Fuad M. Alzahrani, Khalid J. Alzahrani, Shankargouda Patil

**Affiliations:** ^1^ Department of Pharmacy Practice, Faculty of Pharmacy, M.S. Ramaiah University of Applied Sciences, MSR Nagar, Bengaluru, India; ^2^ Department of Pharmacognosy, Faculty of Pharmacy, M.S. Ramaiah University of Applied Sciences, MSR Nagar, Bengaluru, India; ^3^ Department of Oral Pathology & Microbiology, Faculty of Dental Sciences, M.S. Ramaiah University of Applied Sciences, MSR Nagar, Bengaluru, India; ^4^ Department of Maxillofacial Surgery and Diagnostic Sciences, Division of Oral and Maxillofacial Pathology, College of Dentistry, Jazan University, Jazan, Saudi Arabia; ^5^ Department of Clinical Laboratories Sciences, College of Applied Medical Sciences, Taif University, Taif, Saudi Arabia; ^6^ Haematology and Immunology Department, Faculty of Medicine, Umm Al-Qura University, AI Abdeyah, Makkah, Saudi Arabia; ^7^ College of Dental Medicine, Roseman University of Health Sciences, South Jordan, UT, United States

**Keywords:** oral cancer, omics, machine learning, diagnosis, prognosis, therapy

## Abstract

Oral cancer is one of the 19most rapidly progressing cancers associated with significant mortality, owing to its extreme degree of invasiveness and aggressive inclination. The early occurrences of this cancer can be clinically deceiving leading to a poor overall survival rate. The primary concerns from a clinical perspective include delayed diagnosis, rapid disease progression, resistance to various chemotherapeutic regimens, and aggressive metastasis, which collectively pose a substantial threat to prognosis. Conventional clinical practices observed since antiquity no longer offer the best possible options to circumvent these roadblocks. The world of current cancer research has been revolutionized with the advent of state-of-the-art technology-driven strategies that offer a ray of hope in confronting said challenges by highlighting the crucial underlying molecular mechanisms and drivers. In recent years, bioinformatics and Machine Learning (ML) techniques have enhanced the possibility of early detection, evaluation of prognosis, and individualization of therapy. This review elaborates on the application of the aforesaid techniques in unraveling potential hints from omics big data to address the complexities existing in various clinical facets of oral cancer. The first section demonstrates the utilization of omics data and ML to disentangle the impediments related to diagnosis. This includes the application of technology-based strategies to optimize early detection, classification, and staging via uncovering biomarkers and molecular signatures. Furthermore, breakthrough concepts such as salivaomics-driven non-invasive biomarker discovery and omics-complemented surgical interventions are articulated in detail. In the following part, the identification of novel disease-specific targets alongside potential therapeutic agents to confront oral cancer via omics-based methodologies is presented. Additionally, a special emphasis is placed on drug resistance, precision medicine, and drug repurposing. In the final section, we discuss the research approaches oriented toward unveiling the prognostic biomarkers and constructing prediction models to capture the metastatic potential of the tumors. Overall, we intend to provide a bird’s eye view of the various omics, bioinformatics, and ML approaches currently being used in oral cancer research through relevant case studies.

## 1 Introduction

Oral cancer is defined as a heterogeneous group of cancers linked to aberrant changes in the oral mucosal lining. The etiology of oral cancer is multifactorial and predominantly attributed to the accumulation of multiple genetic mutations in the oral epithelial cells (1). It is a substantial public health issue, with a high mortality rate. The major concerns with respect to oral cancer from a clinical standpoint include delayed diagnosis, rapid disease progression, resistance to various chemotherapeutic regimens, and aggressive metastasis which pose a substantial threat to prognosis (2). Understanding the key molecular mechanisms and drivers for drug resistance, disease progression, and metastasis is vital to overcome these problems and improve patient care (3, 4). In the current technology-driven era, the utilization of omics is of paramount importance to address these clinical setbacks (5).

**Figure 1:**
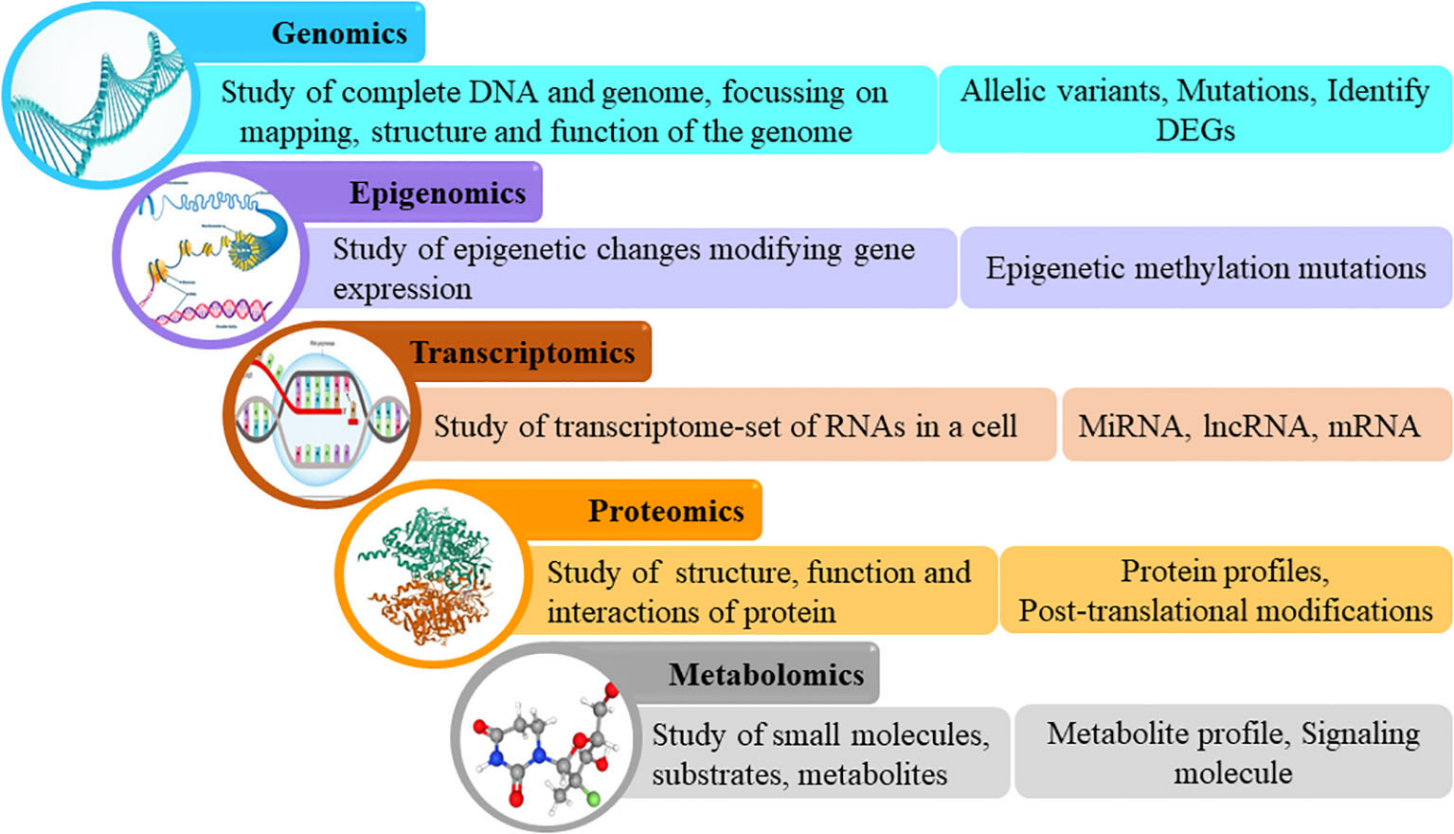
Omics cascade.

Omics leverages big data from genomics, transcriptomics, proteomics, and metabolomics to aid in understanding the molecular mechanisms of the disease on the basis of genes, RNA, proteins, and metabolites respectively (6). In recent times, a multiomics approach, which refers to the use of a combination of these omics strategies, is being employed to simultaneously analyze these biological molecules and their interactions (**Figure 1**). Untangling omics data has the potential to reveal key factors associated with the development and progression of the disease, as well as the response to treatment on a molecular level in order to capture a comprehensive view of the mechanisms underlying the various clinical facets of the disease (5, 7).

**Figure 2:**
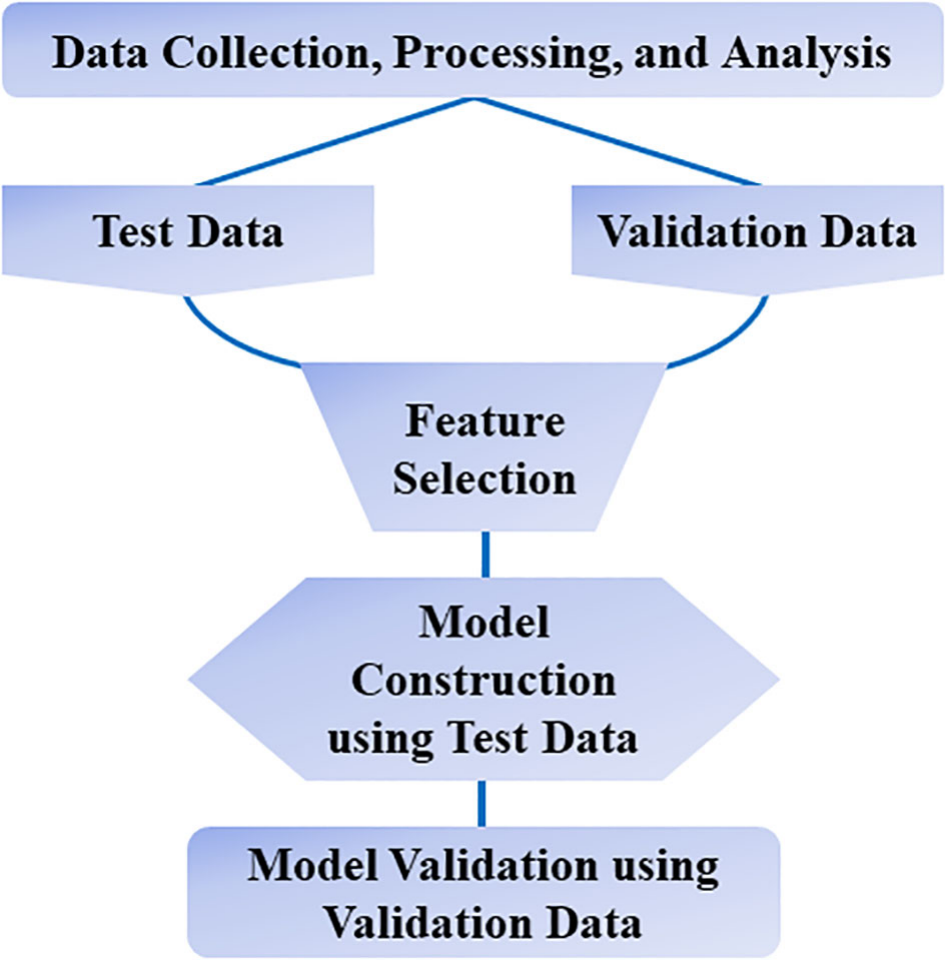
Machine learning workflow.

Further, Artificial Intelligence and Machine Learning (ML) techniques (**Figure 2**) are also being interlaced with omics strategies to shed light on these molecular mechanisms and provide insight into the genetic and molecular abnormalities that are at play in oral cancer. ML strategies enhance the opportunities to discover novel predictive models for diagnosis and prognosis, as well as facilitate the development of personalized treatment strategies based on the patient’s characteristics. Bioinformatics is yet another data-driven field, adopted to explicitly integrate and interpret omics data (8, 9).

The need of the hour in oral cancer research is thus to overcome the aforesaid clinical roadblocks by reconnoitering avant-garde strategies and amalgamating multiple technologies. In this review, we intend to present a bird’s eye view of the various omics, bioinformatics, and ML approaches currently being used in oral cancer research through snapshots from case studies curated to highlight the research methodologies and their clinically relevant outcomes.

## 2 Omics in diagnosis: revelation of novel diagnostic parameters for oral cancer through omics mining

### 2.1 Unearthing candidate diagnostic biomarkers for oral cancer - a multi-omics approach

Wan et al., 2021 (7) employed an integrated approach to explore novel DNA methylation-regulated genes with promising clinical applicability in oral cancer. A combination of RNA sequencing technology along with multiple omics approaches such as transcriptomics and methylomics was used. Transcriptomics refers to the study of RNA to identify changes in the expression of specific genes (10), while methylomics refers to the study of a type of epigenetic modification to the genetic material of a cell involving methylation. Methylation is an essential process of gene regulation, however, abnormal methylation can levy detrimental effects and lead to neoplastic conditions (11).

Initially, transcriptomic data was retrieved from 17 paired samples of oral cancer and normal adjacent tissue by RNA sequencing, following which the data was normalized and processed. Next, a multifactor analysis was carried out using the DESeq2 R program package to identify significant Differentially Expressed Genes (DEGs) using preset criteria. As a result, 2,211 down-regulated and 778 up-regulated genes were captured.

Subsequently, methylomic data was obtained by analyzing the datasets, GSE38532 and GSE46802, from the National Center for Biotechnology Information (NCBI) Gene Expression Omnibus (GEO) database (12). The Differentially Methylated Genes (DMGs) from these two datasets were extracted using the GEO2R software. By integrating the above transcriptomic and methylomic data, a multi-omics analysis was performed using the VennDiagram package of the R program. The overlapping genes between DMGs and DEGs were studied to excavate the aberrantly Methylation-regulated and Differentially Expressed Genes (MeDEGs), which resulted in the identification of 170 down-regulated genes exhibiting a hyper-methylated and 56 up-regulated genes demonstrating a hypo-methylated pattern. Subsequently, the biological functions of these MeDEGs were analyzed using Metascape (13). Biological processes like leukocyte proliferation, response to bacteria, and calcium homeostasis were affected by hypo-methylated up-regulated genes, whereas hyper-methylated down-regulated genes affected the muscle and neuronal system, among others.

The Search Tool for the Retrieval of Interacting Genes (STRING) (14) database was employed to elucidate the Protein-Protein Interaction (PPI) networks pertinent to hyper-methylated down-regulated and hypo-methylated up-regulated genes. Evaluation of these two networks was done by various calculation methods to identify the top ten genes and the common genes in all methods were considered potential hub genes. *CD80*, *CDSN*, *CTLA4*, *GRP29*, *PI3*, and *TNFSF11* were identified from the hypo-methylated network and *ACTN2*, *ISL1*, *MYH11*, *MYOD1*, and *PAX7* from the hyper-methylated network (**Table 1**).

**Table 1:** List of identified methylation regulated differentially expressed genes (7).

Identified Hub Gene	Description	log_2_FC ^a^	Methylation Site (Probe ID)
Upregulated Hypomethylated Genes			
CTLA4	Cytotoxic T-lymphocyte associated protein 4	2.23	cg08460026
GPR29	C-C motif chemokine receptor 6	2.55	cg05824215 cg13615963
TNFSF11	TNF superfamily member 11	3.28	cg21094154 cg24222324
CD80	CD80 molecule	2.54	cg06509940 cg21572897
CDSN	Corneodesmosin	7.20	cg08424423 cg24735489
PI3	Peptidase inhibitor 3	4.28	cg09462575 cg02442161
Downregulated Hypermethylated Genes			
ACTN2	Actinin alpha 2	-8.15	cg16853982 cg21376883
MYOD1	Myogenic differentiation 1	-6.09	cg16519321 cg07271264 cg18555440 cg24322623
ISL1	ISL LIM homeobox 1	-3.74	cg26896762 cg21410991
MYH11	Myosin heavy chain 11	-2.92	cg17880199 cg15488251
PAX7	Paired box 7	-6.31	cg11428724 cg07536847

a log_2_FC, log_2_ transformed fold change.

**Figure 3:**
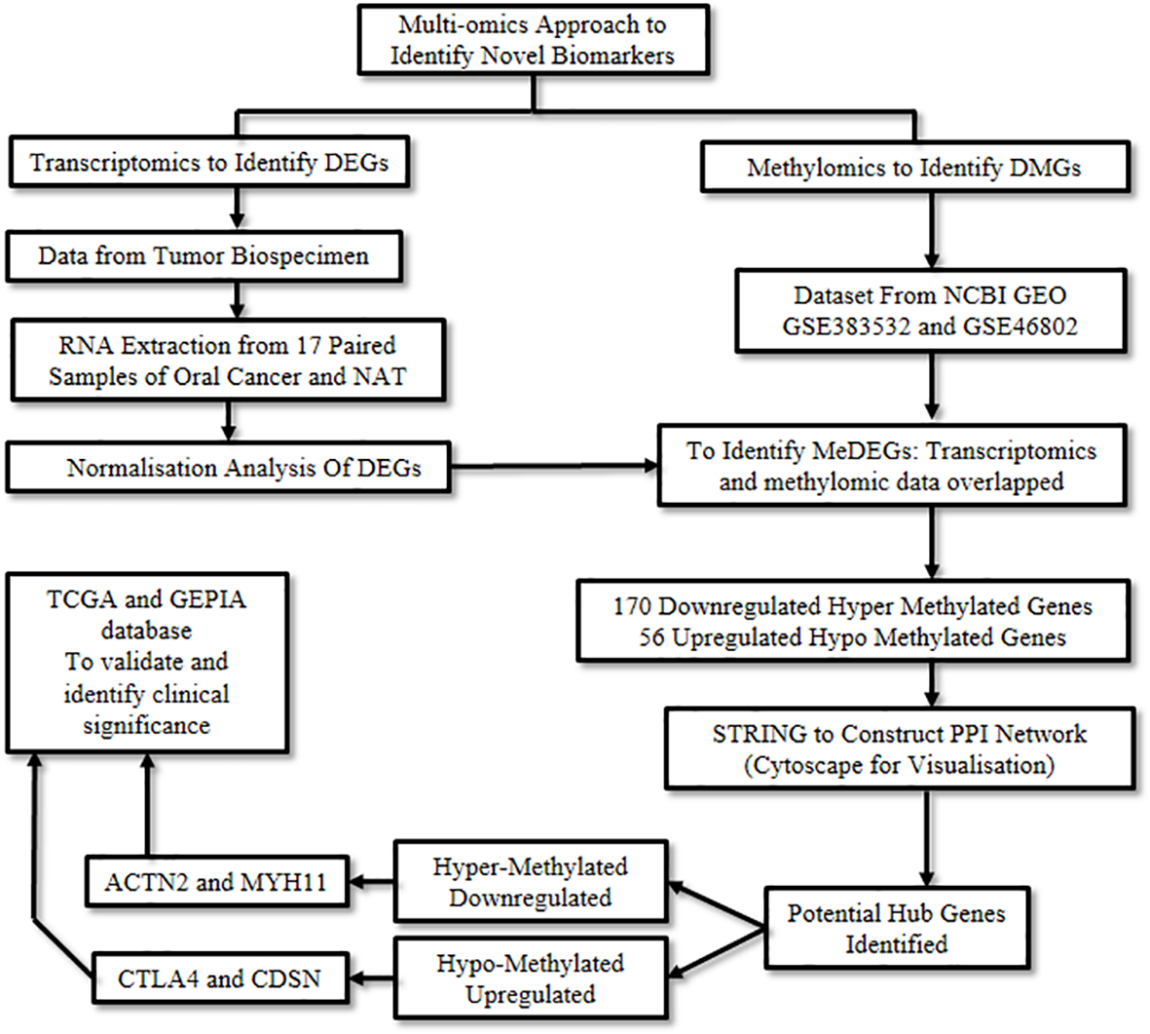
Capturing aberrant epigenetic modifications to aid in oral cancer diagnosis.

Validation of these hub genes was carried out by correlating the expression levels in the disease and the survival effect using the Gene Expression Profiling Interactive Analysis (GEPIA) tool to appraise the clinical importance of the aforesaid hub genes in oral cancer. Four genes were identified to be significantly dysregulated: *CDSN* and *CTLA4* were up-regulated, while *MYH11* and *ACTN2* were down-regulated. In addition, the prognostic significance of the aforementioned 11 hub genes was explored by investigating the link between the degree of expression of each gene and the Overall Survival (OS) of the Head and Neck Squamous Cell Carcinoma (HNSCC) cases using the Kaplan Meier plotter. Herein, HNSCC cases were taken from the GEPIA and The Cancer Genome Atlas (TCGA) database instead of oral cancer cases, due to the unavailability of an independent oral cancer category, however, oral cancer constitutes approximately 95% of the HNSCC cases and was thus considered as a suitable substitute. The degree of expression of the following four hub genes: *CTLA4*, *GPR29*, *ISL1*, and *TNFSF11*, displayed substantial association with the clinical outcome. Amongst these, longer OS was associated with increased expression of *CTLA4*, *GPR29*, and *TNFSF11*, the hypo-methylated up-regulated hub genes, while shorter OS was significantly associated with reduced expression of *ISL1*, the hyper-methylated down-regulated hub gene (**Figure 3**).

In comparison to other epigenetic alterations, DNA methylation is crucial as it is one of the earliest detectable carcinogenic changes and is reasonably reversible by chemical treatment. This research is particularly beneficial to gain insights into the clinical profile of DNA methylation and its impact on gene expression and biological pathways. Aberrant DNA methylation plays an imperative role in oral carcinogenesis, thus highlighting the significance of the study. This multi-omics analysis provides an array of biomarkers for oral cancer which can be instrumental for understanding the molecular mechanisms of the disease and aid in diagnosis.

### 2.2 Intercalating ML and omics - unveiling the diagnostic potential of long non-coding RNA biomarkers in oral cancer

Messenger RNA (mRNA) plays a major role in the translation process and is hence considered one of the hallmark features of mutations or translational inconsistencies in cancer. Similarly, lncRNAs act as critical regulators for assessing the metastatic potential of cancer. The multifaceted role of RNAs in diagnosis, prognosis, and therapeutics of various cancers has been widely studied and explored (15). A study conducted by Yang et al., 2020 (16) focused on identifying the markers associated with the diagnosis and prognosis of oral cancer at a molecular level. Herein, ML and modeling techniques were adopted to explore the patterns in gene expression profiles, particularly the Differentially Expressed mRNAs (DEmRNAs) and Differentially Expressed lncRNAs (DElncRNAs) in order to signify their role as biomarkers in oral cancer.

Initially, relevant RNA-seq and clinical data of 331 oral cancer cases and 44 normal controls were downloaded from the TCGA database and cleaned. The data was then screened with strict parameters to identify DEmRNAs and DElncRNAs in the disease group compared to the control group, using the R package from DESeq2. This step resulted in the identification of 1,114 DEmRNAs of which 345 were up-regulated and 769 down-regulated, and 156 DElncRNAs of which 86 were up-regulated and 70 were down-regulated.

Next, to identify optimal diagnostic lncRNA biomarkers, ML approach was used. Initially, the LASSO algorithm was used along with data dimension reduction to perform the feature selection procedure which resulted in the selection of 30 diagnostic DElncRNAs. The importance of these was then ranked from large to small based on Mean Decrease Accuracy by the Random Forest algorithm. This was then followed by a 10-fold cross-validation analysis to identify the optimum number of features, which was found to be 15. Thus, the top 15 DElncRNAs were chosen as optimal diagnostic lncRNA. Finally, these 15 DElncRNAs were used to establish the classification models, i.e., Random Forest package, Support Vector Machine (SVM), and Decision Tree. The diagnostic ability of these models and the lncRNAs were evaluated based on Receiver Operating Characteristic (ROC), Area Under Curve (AUC), sensitivity, and specificity. The highest AUC was recorded for the Random Forest Model, followed by the SVM model and then the Decision Tree model. The sensitivity of the three models followed a similar trend, while the specificity was similar for the Random Forest Model and the SVM model, and lowest for the Decision Tree model.

Further, a correlation analysis between the 15 lncRNAs and clinical features such as tumor grades was performed via a boxplot to further investigate their diagnostic potential. It was found that irrespective of the tumor grades, the expression of these lncRNAs was significantly lower in normal controls versus oral cancer cases. Additionally, to evaluate their prognostic value a survival analysis was performed, which demonstrated a significant association between FOXD2.AS1 and survival rate, thus indicating its potential as a prognostic marker.

**Figure 4:**
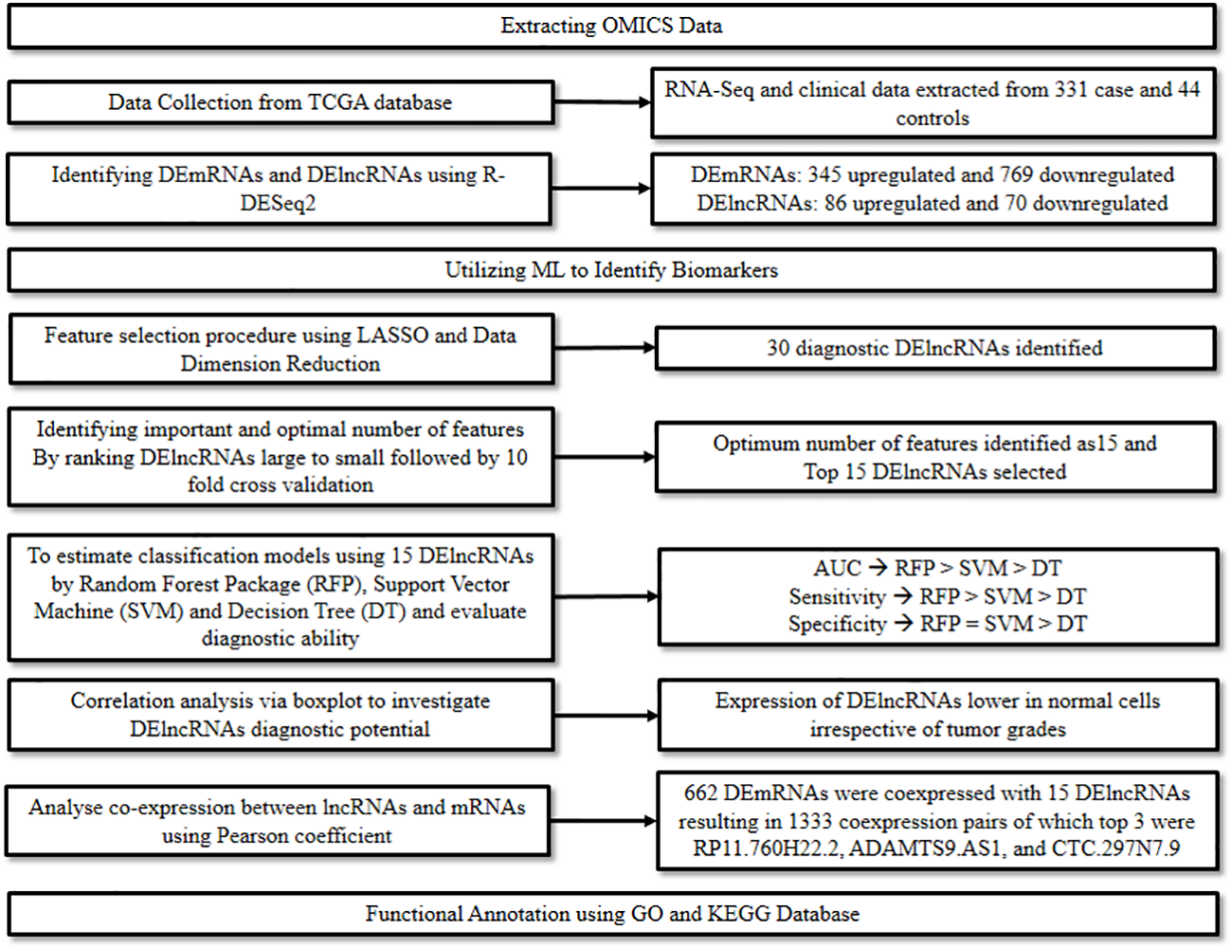
Combining ML and omics to unveil the diagnostic potential of lncRNA biomarkers in oral cancer.

Finally, a co-expression analysis between the lncRNAs and mRNAs followed by a functional analysis was performed to predict the functions of the optimal diagnostic lncRNAs. The co-expression analysis performed using Pearson’s correlation coefficient resulted in the identification of 662 DEmRNAs co-expressed with the 15 DElncRNAs. RP11.760H22.2, ADAMTS9.AS1, and CTC.297N7.9 were discovered as the top three DElncRNAs having the highest numbers of co-expressions with DEmRNAs. The functional analysis was performed for the 662 DEmRNAs using Gene Ontology (GO) and Kyoto Encyclopedia of Genes and Genomes (KEGG). In the GO analysis most DEmRNAs were significantly enriched in the following categories: “signal transduction” in biological process; “cytoplasm” in cellular components; and “protein binding” in molecular function. In the KEGG analysis, the most enriched pathways included the “Focal adhesion” pathway, followed by “ECM-receptor interaction” and “Pathways in cancer” (**Figure 4**). The study thus highlighted the potential of lncRNAs to diagnose oral cancer and revealed the various pathways involved in oral cancer.

### 2.3 Omics in noninvasive biomarker identification and differential diagnosis – amalgamating salivaomics and ML to distinguish oral cancer from oral potentially malignant disorders (OPMDs)

In recent times, it has been found that salivary components, such as proteins, mRNA, and metabolites, are significantly altered in response to certain disease states, including both oral and systemic diseases. Hence, saliva can be used as a source for these biochemical components, which have diagnostic and prognostic capabilities and may act as biomarkers. Approaches that make use of saliva as a source for biochemical data can be termed as salivaomics. This field is increasingly gaining traction due to the ease of collection, storage, and noninvasive features of saliva sampling (17). Similarly, ML techniques are progressively gaining popularity in healthcare diagnostics and the development of assistive diagnostic platforms due to their impressive performance.

In this study, Adeoye et al., 2022 (18) have attempted to combine the principles of ML and salivaomics to optimize the robustness of salivary markers in detecting oral cancer using a ML-supported liquid biopsy platform. In particular, this study utilized genome-wide methylation analysis methods to reconnoiter noninvasive salivary methylome biomarkers and differentially diagnose oral cancer from OPMDs. Further, ML-based platforms were adopted to optimize the role of the shortlisted biomarkers in the diagnosis of oral cancer.

Initially, to evaluate and endorse methylome biomarkers in oral cancer and OPMDs, adequately defined oral cancer and OPMD (Oral Lichen Planus (OLP), erythroplakia, erythroleukoplakia, leukoplakia, and oral submucous fibrosis) cases were selected and their saliva samples were collected. For biomarker discovery and ML analysis, the outcome status for the first 50 patients was discerned. This was followed by randomly selecting 33 samples from this pool for further Reduced-Representation Bisulfite Sequencing (RRBS) to analyze the genome-wide methylation profiles. Post RRBS, differential methylation analysis was performed using Methylkit (19) to capture the Differentially Methylated CpG sites (DMCs) and small-sized Differentially Methylated Regions (DMR) based on the mean methylation percent difference. Overall, 1,745 DMCs were recognized for oral cancer relative to the OPMDs, of which 854 were hyper-methylated and 891 were hypo-methylated. Similarly, of the total 105 DMRs identified in oral cancer patients, 62.9% were hyper-methylated regions and the rest were hypo-methylated regions. Subsequently, the DMCs and DMRs underwent functional annotation and pathway enrichment analysis. The Genomation and Hypergeometric Optimization of Motif Enrichment software was used to functionally annotate the DMCs and DMRs on the basis of their location with respect to genes and CpG islands. This showed that the majority of the DMCs were predominantly enriched in the intergenic regions, 9.4% in CpG islands, and 17.9% in CpG island shores. For pathway enrichment analysis, GO terms related to biological processes, and KEGG pathways were used. For DMCs, important biological processes such as regulation of apoptosis, ion transport, and synapse organization among others, were enriched in GO. KEGG pathway implicated endocytosis, PI3K-Akt, Ras, and Rap1 pathways as the most enriched pathways in oral cancer. Similarly, the 84 DMRs that were enriched in 47 genes, when subjected to the GO-biological processes analysis led to the identification of biological processes such as chromatin organization, pattern specification, and cell-cell adhesion, among others. KEGG analysis also implicated various cancer-related pathways associated with five DMR-associated genes.

Subsequently, to construct predictive ML models to differentiate oral cancer from OPMDs, the percentage methylation for all identified DMCs and DMRs was retrieved. Initially, utilizing each DMC and DMR as a predictive feature, a ML model was created using the following techniques: Adaptive Boosting, Extremely Randomized Trees (ExtraTrees), Decision Tree, Gradient Boosting Machines, k-Nearest Neighbors, Linear and Radial Basis Function, Random Forest, and SVM. To enhance the feasibility and clinical applicability by selecting the most optimal DMCs and DMRs, feature selection using three techniques (ANOVA, MRMR, and LASSO) was performed. After experimenting with various permutations and combinations of the feature selection technique-based DMC and DMR sets and the various ML model techniques, the best performing models were identified. Out of the different DMC-based models, a combination of the common DMCs chosen from all three feature selection techniques and the ML models based on Linear SVM and ExtraTrees exhibited superiority in performance metrics. On the other hand, in the case of DMR-based models, it was found that using a combination of the LASSO-selected DMRs instead of the common DMRs to develop the models showed better outputs. Finally, DMC hypo-methylation of *FGF4* and the LINC00461 associated DMR were found to be the highest significant features for the delineation of oral cancer and OPMDs.

This research demonstrated the viability of using genome-wide methylation analysis techniques to aid in discriminating the features of oral cancer from OPMDs, suggesting its translational potential in a clinical setting.

### 2.4 Omics in oral cancer classification - an attempt to stratify genes based on molecular subclasses and disease stages via bioinformatics

The objective of the study piloted by Shafana et al., 2021 (20) was to identify the genetic markers of oral cancer and classify them not only on a molecular basis but also on tumor stages. The study attempted to develop a novel methodology for this classification and aid in the identification of molecular subclasses of oral cancer at various stages of the disease. This could potentially improve the scope for individualized therapy and identification of the cancer in an early curable stage. The study can be effectively parsed into three sections for ease of understanding. Firstly, DEGs, that is, over and under-expressed genes, were identified, followed by the categorization of the genes based on molecular subclasses, and finally, classification based on the tumor stages, particularly early and late stages.

To identify DEGs, gene expression profiles of 48 oral cancer cases and 8 healthy controls were accessed via the GSE51010 dataset from the NCBI GEO database (12). The data was retrieved and processed using the R programming tool via Bioconductor packages (GEOquery, Biobase, and affy) (21). Post-processing, the data was subjected to cross-platform normalization and underwent noise removal. Subsequently, the DEGs were identified from the panel of significant genes based on stringent cut-off criteria to convert them as gene identifiers by utilizing AnnotationDbi.

To capture molecular subclasses of oral cancer, the ConsensusClusterPlus package was employed to cluster the identified genes and further consensus Cumulative Distribution Function curve analysis led to the identification of 5 subclasses.

Further, to stratify the genes on the basis of early and late tumor stages, the study leveraged clinical data from the datasets and developed a gene co-expression network. A gene co-expression network is developed on the grounds of a similarity matrix with regard to gene expression profiles of many genes, and the genes exhibiting high similarity are correlated and suggested to have similar functionalities. This function is used to associate gene expression with phenotypic traits (22). In this study, based on the clinical data from the datasets, the genes were categorized as either early or late stage. The CoExpress software and the Expression Correlation plugin of Cytoscape (23) software was used to understand the similarity network and to develop the gene co-expression network for early and late stage-specific genes separately based on the correlation between the genes.

Additionally, pathway enrichment analysis was carried out with the help of the Database for Annotation, Visualization and Integrated Discovery (DAVID) tool (24) to excavate vital proteins that play a role in oral cancer. The analysis also aided in the identification of the most influenced canonical pathways, diseases and disorders, molecular and cellular functions, and the transcriptional regulators from the genes that have been categorized depending on the subclasses and stages respectively in the previous steps. This led to the identification of Integrin (*ITGB4*) as an underexpressed gene, its main role being focal adhesion which is important for regulatory signal transmission. Additionally, Serglycin (*SRGN*), a gene with apoptotic function was found to be down-regulated in oral cancer patients, and finally, *GPX2*, a gene responsible for detoxification and antioxidant activity was found to be up-regulated.

Ultimately, the results of the study were validated against the list of marker genes that were previously identified from the Catalogue Of Somatic Mutations In Cancer (25) and NCBI database (12). It was found that most of the genes identified in this study coincided with that of previously identified genes. For example, *TAGLN2*, *CCND2*, and *CCL8* were identified in the study and were previously well-established as tumor suppressor genes. Thus, adding credibility to the current novel methodology and other results of the study.

Successful translation of these discoveries could lead to well defined molecular subtypes of oral cancer and to the revelation of stage-classified genes that can aid in early diagnosis, patient selection, and paving the way for the design of suitable therapeutic protocols.

### 2.5 Excavating crucial oncogenic signatures overlapped in OLP and oral cancer from genomic data

OLP is an oral inflammatory condition with the potential to progress into oral cancer. The study conducted by de Lanna et al., 2022 (26) aimed to highlight the similarities between OLP and oral cancer with regard to gene expression profiles and their roles in inflammatory processes associated with malignant transformation. Additionally, the study focused on identifying new targets and pharmacotherapeutic options to potentially reverse the malignant changes.

Gene expression data from three mRNA microarray datasets consisting of an array of samples derived from oral cancer, OLP, and healthy controls, were accessed via NCBI GEO (12) using the GEOquery R package (27). Similarly, relevant expression data from the TCGA and other GEO microarray datasets were also retrieved to validate the results. The data obtained was preprocessed, integrated, and normalized. Post this, differential expression was analyzed using the limma R package (28) to identify DEGs in OLP, early-stage oral cancer, and advanced oral cancer in comparison with healthy controls. This led to the revelation of 107 DEGs associated with OLP, 331 with early stage oral cancer, and 282 with advanced oral cancer. Of these, a total of 35 genes were found to be overlapping between all three conditions and these were further subjected to non-supervised clustering. As a result, all the samples of OLP clustered with oral cancer samples, predominantly in the early oral cancer stage. Some of the DEGs common to OLP and oral cancer include *KRT4* (down-regulated) and *KRT16*, *KRT17*, *KRT10*, and *KRT75* (up-regulated). These results, when validated using the TCGA data and independent microarray GEO datasets, were found to be consistent.

Subsequently, Gene Set Enrichment Analysis (GSEA) (29) and Over-representation Analysis were performed using the WebgestaltR package (30) and ReactomePA R package (31) respectively, for various sets of OLP and oral cancer genes to identify significant pathways. Pathways related to immunity such as antigen presentation, antimicrobial peptides, complement, and interleukin-10 (IL-10) signaling, alongside other pathways related to extracellular matrix (ECM), non-integrin membrane-ECM interactions, and keratinization were enriched in OLP. Similarly, in oral cancer (early and advanced stage), pathways related to interferon signaling, keratinization, and multiple interleukin, among others, were found to be enriched. Further, it was found that the main pathways enriched in both OLP and oral cancer (either early or late stage), were “antigen presentation pathway”; “formation of the cornified envelope”; “antigen processing cross-presentation”; “interleukin-10 signaling”; “collagen chain trimerization”; “neutrophil degranulation” and “non-integrin membrane-ECM interactions”.

Immune infiltration cell analysis was done to estimate the vital proportions of cells infiltrating both the conditions by using the CIBERSORTx tool (32) and the tool’s genetic signatures available for 22 cell types. This includes various B cell types, T cell types, natural killer cells, dendritic cells, macrophages, mast cells, neutrophils, monocytes, and eosinophils among others. As a result of this analysis, it was found that, when compared to the normal oral mucosa, the levels of activated natural killer cells in OLP and oral cancer samples were significantly lower. Also, significantly higher proportions of M0 and M1 macrophages and CD8+ T lymphocytes were found in advanced-stage oral cancer when compared to OLP. In contradiction, reduced proportions of resting naïve B cells, Mast cells, and monocytes were observed in both stages of oral cancer relative to normal oral mucosa and OLP. Further, gene signatures related to non-pathogenic and pathogenic Th17 cells were explored by employing a 33-gene signature panel based on previously characterized Th17 phenotypes. Oral cancer displayed predominant gene expression pertinent to the pathogenic Th17 signature, while non-pathogenic Th17 cell profile expression was high in OLP and control mucosa. In OLP, pathogenic signaling-related genes such as *IL1B*, *LTA*, *LTB*, *CTSC*, *HIF1A*, and *TGFB3* were differentially modulated. Further, higher expression of *CTSC*, *HIF1A*, and *IL1B* was observed in both stages of oral cancer. These findings were in line with that of the validation datasets.

Next, to analyze the connection strength between genes showing similar expression patterns and identify co-regulated genes involved in the cancerization of OLP, gene co-expression modules were developed with the help of the Weighted Gene Co-expression Network Analysis R package (33, 34). 12 such co-expression modules were developed by analyzing 15,000 genes. Out of the 12 modules, a significant positive correlation with OLP was observed in only one. In this module, to better understand the interactions of the genes, a PPI network was constructed in the STRING (14) database which led to the identification of 11 hub genes. Further FDA-approved anticancer drug-gene interactions and gene categories for the hubs were determined using the Drug-Gene Interaction Database (DGIdb) online tool (35). This led to the identification of 15 transcription factors, 112 genes in the druggable genome, and 6 genes that were clinically actionable. Subsequently, 89 drugs that interacted with 3 of 11 hubs were selected. Further study of the hub gene expressions revealed that *PI3* was the only gene that was significantly up-regulated in all the conditions while *DSC1*, *DSG1*, *IVL*, and *PKP* did not demonstrate significant differences in expression when compared to the control. Three of the 11 hub genes had previously known drug-gene interactions, that is, *PI3* and *DSP* which were up-regulated in OLP and oral cancer, and *IVL* which was up-regulated in OLP and early-stage oral cancer.

Subsequently, to identify drugs with the ability to reverse the expression signatures of the selected overlapping DEGs, the L1000 Characteristic Direction Signature Search Engine tool on the Library of Integrated Network-Based Cellular Signatures Program platform was used (36, 37). Additionally, the drugs identified were evaluated using the repoDB database (38) to assess the repositioning possibilities. As a result, PI3K/mTOR pathway inhibitors were found to be one of the most represented categories of drugs including agents like Torin-2, GSK 1059615, INK-128, and GDC-0980, among others.

## 3 Omics in disease management: unearthing potential therapeutic strategies to confront oral cancer by exploring omics big data

### 3.1 Reprofiling CYP 450 inhibitors against oral cancer - a contemporary drug discovery endeavor

Oral cancer is characterized by several hallmark features amongst which up-regulation of *Akt1* and *Akt2* is considered significant (39, 40). On the other hand, the *MAOB* gene is reported to be down-regulated in cancer progression (40). In pursuit of candidate/lead molecules for oral cancer, it is hypothesized that chemical entities inhibiting the overexpression of Akt1 and Akt2 without suppressing the expression of MAOB may demonstrate promising therapeutic potential. Based on the above concept, Siam et al., 2021 (41) leveraged the molecular docking technique to explore potential leads with the aforementioned properties. This research group believed CYP inhibitors to be an ideal option for modulating Akt1 and Akt2 proteins with minimal or no impact on MOAB expression.

Initially, intensive literature mining was carried out to capture potent Cytochrome P450 (CYP3A4) inhibitors derived from herbs. Structures of the resultant molecules were obtained from DrugBank and PubChem in SDF format and concurrently converted using Open Babel into PDB format (42–44). This served as a test set for molecular docking studies, while Capivasertib and A-443654 were selected as reference inhibitors to dock against Akt1 and Akt2 respectively. The 3D structures of Akt1 and Akt2 were retrieved from Research Collaboratory for Structural Bioinformatics-Protein Data Bank and curated using PyMOL (45, 46). The obtained proteins and ligands were docked using AutoDock Vina (47) and the protein-ligand interactions were visualized using Discovery Studio. RAMPAGE, an online server was used to analyze the Ramachandran plot, while admetSAR 2.0 (48) offered insight into the pharmacological properties of the studied drugs.

Docking studies revealed that a few CYP inhibitors exhibited higher binding affinities than reference Akt1 and Akt2 inhibitors. Amongst these CYP inhibitors, galuteolin and linarin were remarkable and were subjected to further analysis. The protein-ligand interaction complexes of the CYP inhibitors, that is, galuteolin and linarin, and reference inhibitors were visualized by superimposing them on the binding pockets of their respective Akt proteins. This revealed that the CYP inhibitors and the reference Akt inhibitors shared the same binding pockets of Akt proteins. Later, respective Akt protein-reference inhibitors complex and Akt protein-CYP inhibitors complex were analyzed to identify the common amino acid residues involved in the interaction. The CYP inhibitors and reference inhibitors had overlapping interacting amino acids, hence the test drugs were assumed to interact with the same protein pocket as the reference drugs. Finally, the ADMET (absorption, distribution, metabolism, elimination, and toxicity) properties of the CYP inhibitors were analyzed and found to be superior when compared to the reference inhibitors in terms of blood brain barrier permeability.

**Figure 5:**
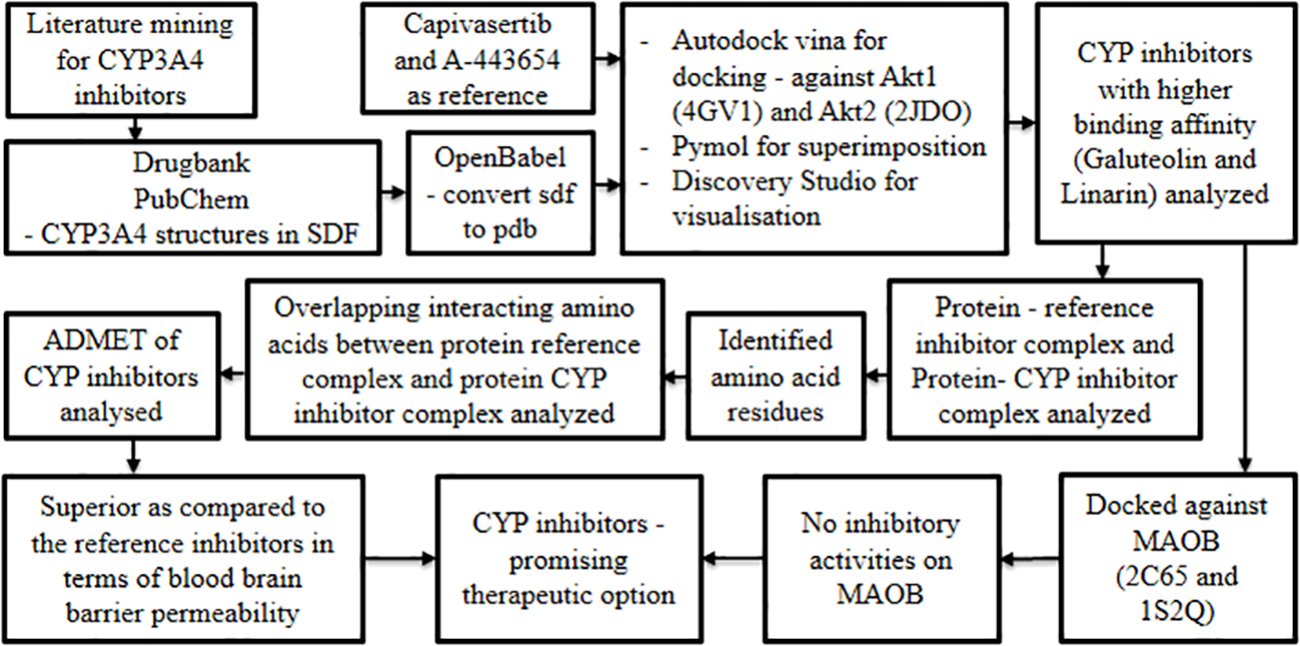
Unveiling the repurposable potential of CYP 450 inhibitors against oral cancer.

After analyzing the Akt inhibitory properties of the CYP inhibitors, their influence on MAOB proteins was investigated. Herein, two PDB structures of MAOB (2C65 and 1S2Q) were docked in rigid mode with the literature-derived CYP inhibitors having Rasagiline as standard using AutoDock Vina (47). This revealed that galuteolin and linarin did not exhibit any inhibitory activities on MAOB (**Figure 5**). The results of this study emphasized the capability of the above CYP inhibitors to be a promising repurposable therapeutic option for oral cancer.

### 3.2 Omics in combating drug resistance - interlacing proteomic and phosphoproteomic data to unearth kinases with druggable potential in chemotherapy-resistant tongue cancer

Tongue cancer is one of the most aggressive and prevalent forms of oral cancer which is associated with rapid progression and poor prognosis. The treatment is dependent on the stage of the cancer, and in cases with locally advanced inoperable tumors, neoadjuvant chemotherapy (NACT) has been identified as a beneficial regimen. However, the success of NACT is limited by the development of resistance, low response rates, and local relapses. George et al., 2022 (49) explored the proteomic and phosphoproteomic profiles of NACT resistant and sensitive tongue cancer patients to identify differentially expressed proteins associated with these properties by using Liquid Chromatography-tandem Mass Spectrometry (LC-MS/MS) techniques. The study aimed to provide a molecular background for the mechanisms of NACT resistance which could potentially aid in overcoming treatment resistance.

Tissue biopsy samples from treatment-naive tongue cancer patients were collected and the tumor composition of the samples was pathologist verified. Following this, all the patients underwent NACT with standard drug dosages for a specified duration. Following the second cycle of chemotherapy, the treatment response was evaluated using Response Evaluation Criteria in Solid Tumors (50) and recorded, based on which the patients were categorized as either responders or non-responders, respectively. Next, the proteome and phosphoproteome of these patients were quantified, using multiplexed Tandem Mass Tags (TMT), Immobilized Metal Affinity Chromatography (IMAC)-based phosphopeptide enrichment, and LC-MS/MS methodologies, to evaluate the protein expression and phosphorylation events associated with resistance (from 4 non-responders) and sensitivity (from 4 responders) to treatment. The data was then processed using the MASCOT and SEQUEST-HT algorithms from the Proteome Discoverer 2.0 platform. The global proteome and phosphoproteome data of the resistant and sensitive patients were compared. From the global proteome data, 7,453 proteins were identified and 7,000 of these proteins that were present in all samples were quantified. From these, 15,440 phosphopeptides were identified and 9,385 were quantified, corresponding to 4,150 and 3,106 proteins respectively, across all the patients. Next, the PhosphoRS algorithm was used to identify serine/threonine/tyrosine phosphorylation sites, and post quantification 9813 serine, 1,266 threonine, and 87 tyrosine phosphorylation sites corresponding to 9,385 phosphopeptides which in turn correspond to 3,106 proteins, were identified.

The proteomic and phosphoproteomic data were then statistically analyzed separately to identify differentially expressed proteins and differentially phosphorylated proteins in the resistant cohort relative to the sensitive cohort. This resulted in the recognition of 59 differentially expressed proteins out of 275 significantly expressed proteins and 126 differentially phosphorylated phosphopeptides corresponding to 98 proteins out of 305 significantly phosphorylated phosphopeptides. These significantly expressed proteins and phosphorylated phosphopeptides separately underwent unsupervised clustering, which led to the identification of unique proteomic signatures in the resistant cohort. Upon further study, it was observed that proteins such as aspartic peptidase retroviral like 1 (ASPRV1), keratin 1 (KRT1), and keratin 10 (KRT10) among others, were highly expressed in resistant patients. Similarly, hyperphosphorylation of various keratins was observed in the phosphoproteomics data of the resistant cohort. KRT10 was observed in the non-responders at the sites S33, S39, S42, S56, S61, S459, S537, and S577, while hyperphosphorylation of KRT1 was observed at the sites of S13, S66, S612, S344, Y639, S618, S609, and S541. Furthermore, a comparison between the differentially expressed proteins and differentially phosphorylated proteins revealed five proteins that showed significant changes in both protein expression and phosphorylation, and 81 proteins that were dysregulated only at the phosphorylation level with no difference in protein expression. These 81 proteins were then used to construct a PPI network in the STRING (14) database.

Pathway enrichment analysis of these differentially expressed and phosphorylated proteins was done using the Reactome analysis tool (51), which revealed distinct pathways. Differentially expressed proteins demonstrated enrichment in pathways such as “interleukin-18 signaling”, “formation of the cornified envelope”, and “keratinization” among others. In contrast, differentially phosphorylated proteins revealed significant pathway enrichment related to the Rho GTPase cycle and its signaling. The resistant cohort of patients was associated with 17 differentially phosphorylated proteins which were implicated in the Rho GTPase cycle. Pathways enriched in both proteomic and phosphoproteomic data included those related to keratinization and formation of cornified envelope.

From these differential phosphorylation events, kinases were investigated and the identified kinases were searched and the KinMap tool (52) was used to construct a kinome map. 191 kinases from various kinase families such as tyrosine kinase, tyrosine-like kinase, and calmodulin/calcium-regulated kinase among others were identified and a kinome map was created. Further, in the resistant cohort hyperphosphorylation of kinases such as Mammalian Ste20-like protein kinase 3 (MST3), Microtubule affinity-regulating kinase 2 (MARK2), and Fibroblast growth factor receptor 3 (FGFR3) was observed. Next, kinase-substrate enrichment analysis was carried out for the differentially phosphorylated proteins to study the molecular regulatory mechanisms involved in NACT resistance. Using the Kinase Enrichment Analysis 3 tool, Mitogen-activated protein kinase 1 (MAPK1), Akt1, and Mitogen-activated protein kinase 3, were predicted as the top three enriched kinases that are involved in hyperphosphorylation among others, while SRC and ABL1 were identified as the top two kinases involved in hypophosphorylation. PhosphoSitePlus (53) was used to identify upstream phosphatases for the downregulated kinases which resulted in the identification of the following upstream phosphatases for the SRC kinase: PTEN, PPP2CA, PTP52N13, PTP1B, PTPRJ, PTPRA, and SHP-2.

Next, the protein expression and mRNA of the predicted kinases were studied using publicly available data from cBioPortal. Using the expression data of 131 tongue cancer patients from the TCGA database, the mRNA expression was examined. Variable expression of the predicted kinases from the current study was also observed in this dataset. Similarly, using the protein expression data of 61 patients from the Reverse Phase Protein Arrays in signaling pathways database the protein expression was investigated and the expression of only the top two kinases were found, that is MAPK1 and Akt1, and these too were variably expressed.

Finally, the Therapeutic Target Database was queried to identify enriched druggable targets from the hyperphosphorylated kinases and predicted kinases for NACT resistant patients. Mainly inhibitors of the enriched kinases identified in the current study were found and were either approved or in clinical trials. For example, MAP3, a kinase predicted to be enriched in resistant patients from this study, currently has 2 antagonists, ravoxertinib and ulixertinib, which are in clinical trials.

### 3.3 Omics complementing surgery - exceptional insight to differentiate oral cancer surgical margins using transcriptomic biomarker signatures

One of the crucial aspects related to the survival of cancer patients post-surgery depends on the degree of excision of the original tumor. The residual dysplasia prevailing in resection margins post-surgery is considered a common attributing factor for the recurrence of the disease.

The significance of whole-transcriptome gene expression and short non-coding RNA profiles in developing distinctive biomarkers to distinguish surgical margins in oral cancer was demonstrated by Fox et al., 2022 (54). Patients undergoing surgical resection for oral cancer were chosen to study the differential gene expression profiling on biopsy samples of the tumor, near margin, and distant margin sites. This study involved enrolling 18 oral cancer patients prior to their surgical intervention, and the oral cancer sites were visualized using Narrow Band Imaging (NBI) and conventional white light illumination.

Later, oral cancer resection was carried out at the level ≥5 mm beyond the surgical margin defined by NBI; subsequently, punch biopsies of 4 mm dimension were obtained from (1) 5 mm away from the limit of tissue abnormality showcased by NBI which is considered as normal (N), (2) 5 mm away from the limit of tissue abnormality observed under white light, which is considered as margin (M) and (3) the primary tumor core (T).

In general, tumor histology was mostly moderate to highly differentiated oral cancer with a single incident of verrucous carcinoma. The margins in independent histopathology were ‘clear’ in all samples except one, and two samples showed margins that were more than 5 mm demarcated from the tumor. Also, one ‘close’ margin was detected at 1 mm. Based on this approach, it was reported that the five-year disease-free survival (DFS) and the local recurrence rate were 84.21% and 5.26% respectively.

In addition to this, mRNA expression was collated from Human Genome U133 Plus 2.0 followed by quality control procedures. The suitable data were subjected to pre-processing steps and normalized by the Guanine Cytosine Robust Multi-Array Analysis (GCRMA) method. Subsequent to this, specific mRNA probes were correlated to their respective genes and the functional annotation of the resultant genes was achieved with the DAVID tool (24). Through this, a total of 4,794 genes were identified by differential gene analysis, and individual comparisons revealed the following number of genes: 4,387 for T *vs*. N, 3,266 for T *vs*. M, and 7 for N *vs*. M. Likewise, miRNA expression analysis showcased 119 differentially expressed miRNA, further, upon individual comparisons 109 miRNAs were observed at T *vs*. N, 81 miRNAs at T *vs*. M and 7 miRNAs at N *vs* M. These findings imply that N samples were comparatively more molecularly distinct from T samples than from M samples, and thus justify the adoption of a complementary optical imaging technique in oral cancer surgical excision.

As a further step, multivariate regression algorithms (sPLS-DA) were performed to identify the key variables unique to each group. The aforementioned task was executed using the mixOmics R package (55) with ‘tune’ function in place to retrieve an array of parameters with a minimal error rate. This method aided in the identification of the classifying gene or miRNA expression signatures that distinguish various tissue zones (N, M, and T). Preliminary modeling yielded 14 optimally performing genes that are highly effective in discriminating T samples, while less effective in differentiating N and M samples. The analysis was repeated without T samples and that resulted in a binomial model consisting of 20 genes. These genes performed well in the discrimination of N and M samples. Therefore, this profile of 20 gene expression biomarkers can be considered to differentiate the more distant normal tissue from the histologically tumor-free margin.

In parallel, a classifying model based solely on miRNA expression was developed using the same approach as previously stated, i.e., multivariate regression methods (sPLS-DA). Intriguingly, the gene expression model outperformed the miRNA expression-based model in terms of classification ability, specifically for the T and M zones.

Additionally, the performance of elastic net regression, which used a gene expression dataset as input, was examined to determine biomarkers and classify samples. Unfortunately, no subset of genes worked best for developing a prediction model for multinomial classification. Adding to this fact, it was also noted that the elastic regression analysis performed inferior to that of sPLS-DA in discrimination.

Furthermore, the classifier genes such as *MYO1B*, *MMP1*, *TNFRSF12A*, *MMP12*, *LAMC2*, *WDR66*, *SLC16A1*, and *PLAU* that were elevated in tumor tissue were identified to generate biomarkers to spot residual abnormalities in the surgical margins. Application of the aforesaid gene signature to classify 18 marginal samples predicted 13 as normal and 5 as tumors. The interesting fact is that some of the forecasts were unambiguous while others were more borderline.

**Figure 6:**
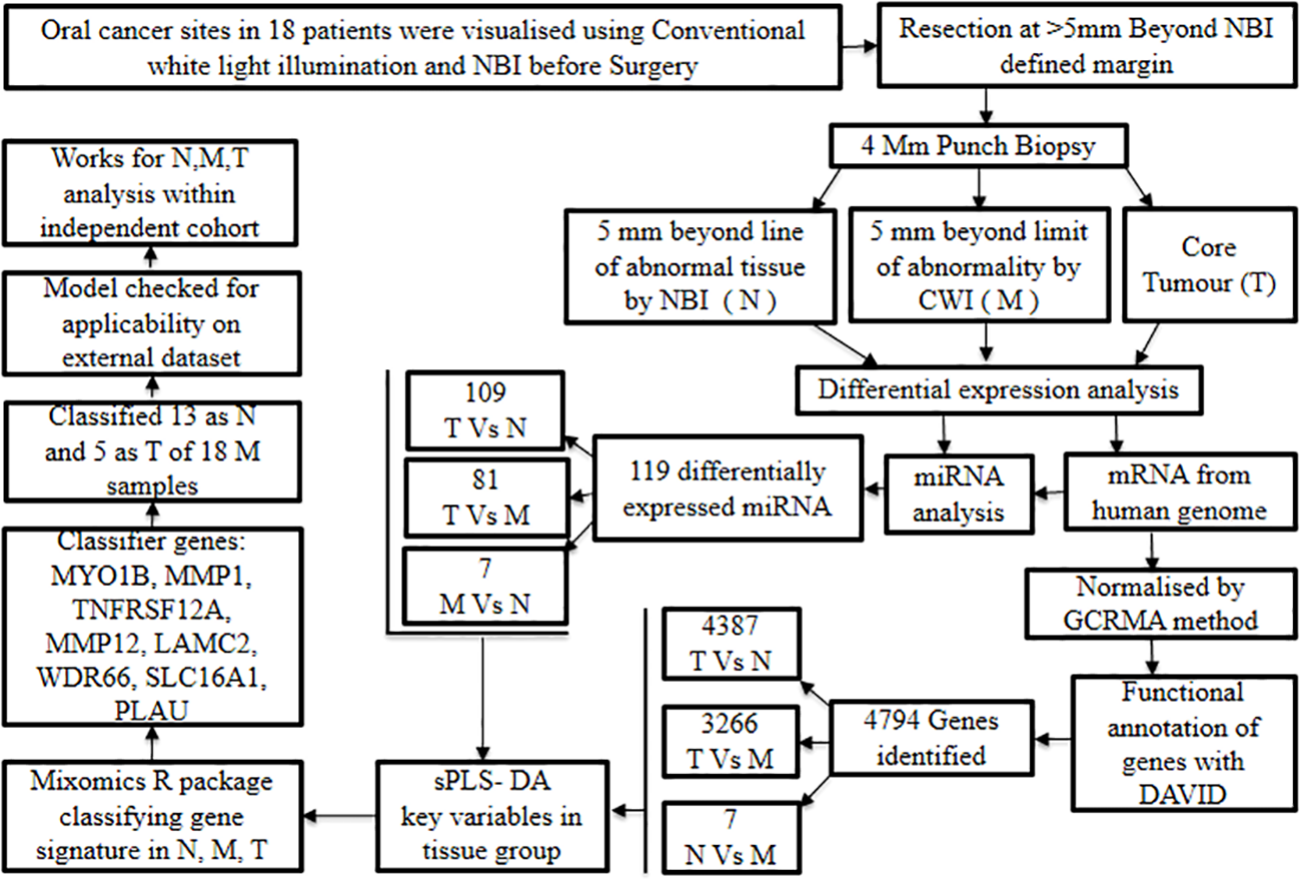
Discerning surgical margin based on transcriptomic biomarker signatures.

In addition, the generated prediction model was tested using an external dataset consisting of N, M, and T samples to evaluate its generalizability and clinical applicability. The model identified 13 out of 49 M samples as tumors with a prediction score of 0.5 or more and the survival analysis failed to demonstrate a significant difference between the groups (**Figure 6**). According to these findings, discrimination signatures were successful in recognizing cancer samples within the independent cohort, while the samples linked to recurrence could not be distinguished using the statistical model signature of abnormality in the margins. In conclusion, the biomarker signatures identified could complement the current surgical and histological approaches in the early detection of tumors in oral cancer with clean surgical margins.

## 4 Omics in prognosis: forecasting prognostic outcomes for oral cancer using omics methodologies

### 4.1 Comprehensive bioinformatics analysis to spot the latent biomarkers of oral cancer associated with progression and prognosis

Oral cancer research is primarily focused on identifying disease-specific genes to comprehend their molecular and biochemical activities alongside their role in intricate interaction networks that influence the development, progression, and prognosis.

To unravel the DEGs in oral cancer, Reyimu et al., 2021 (56) mined three mRNA expression datasets: GSE9844 encompassing 26 tumor and 12 normal tissues, GSE30784 containing 167 tumor and 45 normal tissues, and GSE74530 comprising six tumor and six premalignant tissues; and one miRNA expression dataset, GSE124566 which included 10 tumor and 10 normal samples. The datasets were analyzed using the R package (limma) (28) with a set screening criteria (adjusted P < 0.05, and log2FC > 1). This revealed the differential expression of 298 genes in oral cancer tissue inclusive of 98 down-regulated genes and 200 up-regulated genes.

Following this, GO functional annotation analysis of DEGs revealed their enrichment in “extracellular structure and matrix containing collagen”, while, pathway enrichment analysis in KEGG confirmed their significant enrichment in IL-17 and PI3K-Akt signaling pathway. Later, using STRING and Cytoscape (23), the genes were subjected to a PPI network as well as miRNA-gene network analysis. The shortlisted central genes were screened based on gene degree, molecular complex detection plugin, and miRNA-gene network.

**Figure 7:**
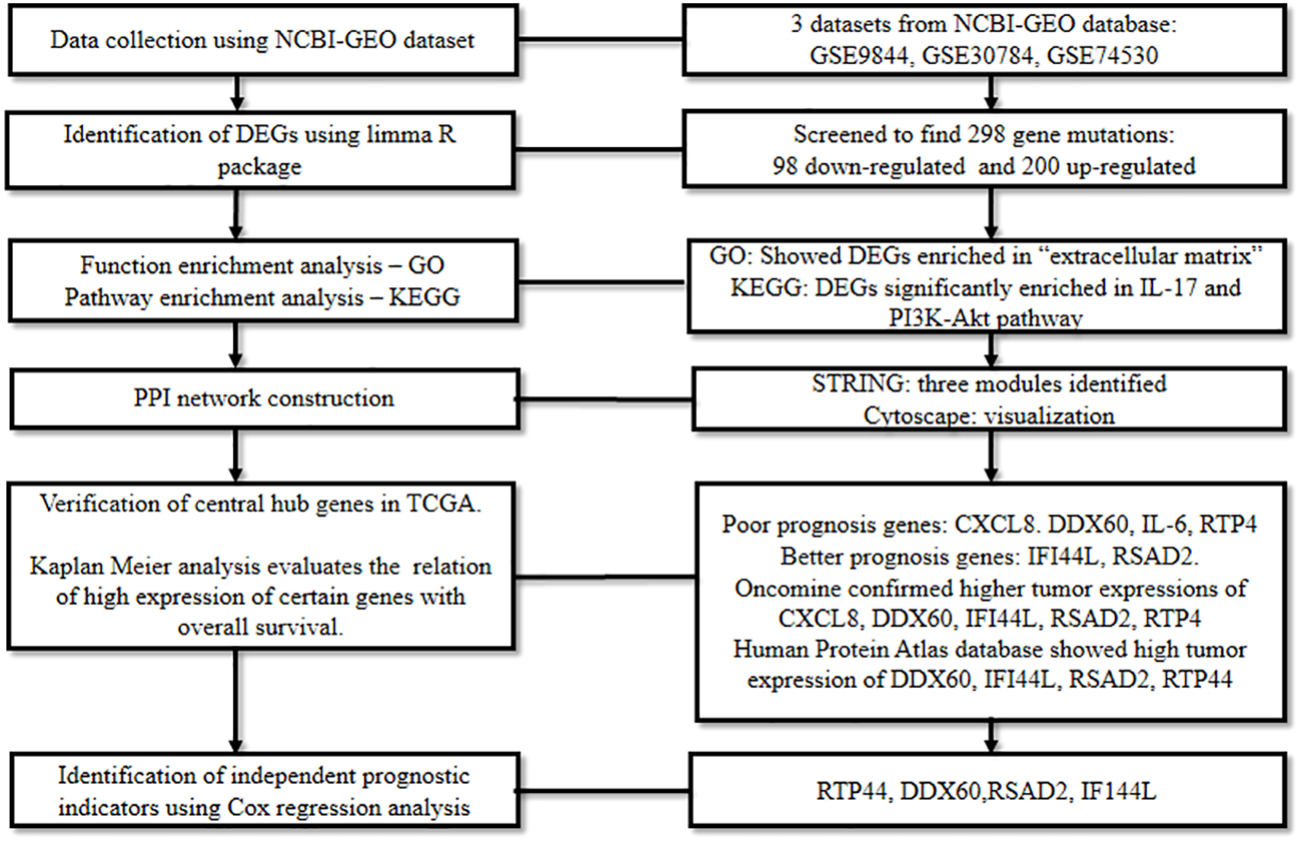
Comprehensive bioinformatics analysis to spot the prognostic biomarkers of oral cancer.

Subsequently, the recognized genes were analyzed for their significance in TCGA and Kaplan Meier data which correlated a high expression of *IL6*, *CXCL8*, *DDX60*, and *RTP4* with poor prognosis in oral cancer patients, while a better prognosis was linked with a high expression of *IFI44L* and *RSAD2*. Raised expression of *CXCL8*, *DDX60*, *IFI44L*, *RSAD2*, and *RTP44* in oral cancer was confirmed by Oncomine (57), and The Human Protein Atlas database (58) demonstrated higher expressions of *RTP44*, *DDX60*, *RSAD2*, and *IFI44L* in malignant tissues. Finally, according to Cox regression analysis, *RTP44*, *DDX60*, *RSAD2*, and *IFI44L* were identified as independent prognostic indicators of oral cancer (**Figure 7**). This research presented a framework of putative biomarkers and pertinent pathways linked with the prognosis of oral cancer.

### 4.2 Construction of ferroptosis-related-lncRNAs-based oral cancer prognostic prediction model

Qiu et al., 2022 (59) identified ferroptosis-related lncRNAs from oral cancer patient data to build a prognostic predictive model.

Firstly, the RNA-seq data of 338 oral cancer patients from the TCGA portal was analyzed using a rank sum test. This resulted in 386 DElncRNAs out of which eight ferroptosis and prognosis-related lncRNAs (AC079921.2, AC099850.3, AC090246.1, FIRRE, AL512274.1, MIAT, LINC01305, and LINC00524) were predicted via univariate Cox survival analysis.

Further, the FerrDb website (60) and literature mining yielded 382 ferroptosis-related genes that encompassed ferroptosis markers along with ferroptosis-suppressing and ferroptosis-inducing genes. Following this, a correlation network was constructed between the acquired prognosis-related lncRNAs derived from the patient data in this study and literature/website-derived ferroptosis-related genes. The constructed network was visualized in Cytoscape (23), which revealed the co-expression of AL512274.1, LINC01305, and AC099850.3, with a comparatively increased number of ferroptosis genes.

The precision of the established model was further assessed through survival curves, ROC curves, and clinical Decision Curve Analysis (DCA), while independent prognostic factors were assessed using univariate and multivariate Cox regression analyses. This determined the risk scores based on the expression levels of risk regression coefficients and ferroptosis-related lncRNAs. Accordingly, the patients were allocated to low or high-risk groups. Subsequent survival analysis revealed that the high-risk group had a lower OS rate when compared with the low-risk group. Survival status together with ROC analysis suggested that the risk model developed, performed well in predicting the patient prognosis.

Factors that could independently predict the prognosis were analyzed by univariate and multivariate Cox analysis. Univariate Cox analysis of patients’ clinical features revealed that age, stage, risk score, and tumor grade varied considerably and were risk factors for oral cancer. Multifactorial Cox analysis indicated that the risk score may independently signify the prognosis of oral cancer.

Concurrent heat map analysis of eight predicted lncRNAs between the low- and high-risk group disclosed that AC090246.1, AC099850.3, and FIRRE were up-regulated, while AL512274.1, AC079921.2, LINC01305, and MIAT were downregulated in the high-risk group, thus confirming the accuracy of the model in predicting oral cancer prognosis.

Followed by this, a DCA curve was plotted by analyzing the association between predicted prognosis and clinicopathological parameters involving ROC of clinical characters and risk score. This analysis also confirmed that the risk score was a more promising prognostic factor than other clinical markers. Further study on the correlation between the risk scores with individual clinical features followed by heat map analysis demonstrated a substantial difference in the T stage of oral cancer between low and high-risk groups. Subsequently, a nomogram was constructed considering the factors such as age, gender, risk score, grade, TN stage, and other prognostic factors to predict the survival rate of a patient using personal data. Intriguingly the foreseen survival rate was very near to the ideal line signifying the accuracy of the prediction made by the nomogram which can serve as a tool for future personalized treatment.

Furthermore, the relationship between the risk model and immune cell infiltration was analyzed to identify the difference in immune-related functions (T-cell co-stimulation, T-cell co-inhibition, CCR, and HLA) between the low and high-risk groups. This revealed a significant difference in immune status between the groups, reinforcing the requisite for customized immunotherapy.

An additional examination of differences in m6A-associated genes and immune checkpoints between the two groups revealed that only 29 checkpoint genes were found to be remarkably differentially expressed between the groups. Additionally, examination of gene expression differences between the two groups revealed that m6A-related gene *YTHDC2* was significantly down-regulated while *HNRNPC*, *ALKBH5*, and *YTHDF1* were significantly up-regulated in high-risk groups.

Later, KEGG analysis uncovered the involvement of almost 24 and 10 active signaling pathways in the low and high-risk group patients respectively. These were linked with metabolic pathways pertinent to purine, pyrimidine, and spliceosome in the high-risk group while, immune-associated biological processes such as the B-cell receptor pathway, T-cell receptor pathway, and FcϵRI pathway in the low-risk group.

Simultaneously, an attempt was made to recognize the potential drugs to target oral cancer, utilizing the up- and down-regulated ferroptosis-related genes via the L1000FWD database (61). This fetched MEK inhibitors, estrogen receptor agonists, and RAF inhibitors among others, as possible reference candidates. Differences in gene enrichment were observed when the cell lines were treated with these molecules. The top ten candidate molecules include KM-03949SC (MEK inhibitor), RJC-00245SC (estrogen receptor agonist), BRD-K82185908 (adrenergic receptor antagonist), KM-00519SC (RAF inhibitor), BRD-K94987138 (histamine receptor antagonist), BRD-K67619794 (histamine receptor antagonist), BRD-K05197617 (EGFR inhibitor), Ivermectin (benzodiazepine receptor agonist), Vemurafenib (RAF inhibitor), and BRD-K03122949 (dopamine receptor antagonist).

Finally, the alteration in the expression of ferroptosis-related lncRNAs with regard to varying clinical characteristics was studied using real-time PCR. This revealed the following findings: (a) AC079921.2, AL512274.1, AC090246.1, AC099850.3, FIRRE, MIAT, LINC00524, and LINC01305 in tumor and normal tissues, (b) AC090246.1 and AL512274.1 in N stage tumors, (c) MIAT and AL512274.1 in the lymphovascular invasion; (d) AL512274.1, LINC01305, and AC079921.2 in different grades; (e) MIAT, AC099850.3, and AL512274.1 expression had a strong correlation with OS rate.

Additionally, expression levels of the selected eight lncRNAs were detected in four pairs of matched oral cancer and adjacent normal tissues. In contrast to nearby normal tissues, oral cancer tissues had greater relative expression levels of AC099850.3, AC079921.2, AC090246.1, FIRRE, MIAT, LINsC01305, and LINC00524, whereas, AL512274.1 had lower relative expression levels. The results were in agreement with those of the model analysis. In conclusion, the developed model may potentially serve as a valuable tool to predict prognosis and explore ferroptosis-related lncRNAs in oral cancer.

### 4.3 Development and corroboration of oral cancer prognostic model based on metabolism-related genes

Zhang et al., 2020 (62) developed a model based on Metabolism-Related Genes (MRGs) to evaluate the prognostic outcome of oral cancer.

Initially, the FPKM normalized gene expression profiles along with clinical information of around 340 cancers and 32 non-cancer samples were retrieved from TCGA oral cancer cohort (training set), in addition to 97 oral cancer samples from the GSE41613 GEO database (12) (validation set). Later, the samples from either source that corresponded to less than 90 day follow-up period were excluded, which resulted in the inclusion of 319 patients from the training set and 94 from the validation set. The metabolic DEGs within cancer relative to non-cancer samples were screened by differential analysis using the limma R package (28). The results showcased a total of 317 MRGs inclusive of 142 down-regulated and 175 up-regulated genes.

Later, univariate Cox regression analysis performed to construct a prognostic signature revealed 12 differentially expressed MRGs that were positively correlated with poor prognosis. These risk genes were considered as possible molecular markers to predict prognosis in oral cancer. On the other hand, Lasso-Cox regression analysis established 11 prognostic gene models (*ADA*, *ADK*, *ATIC*, *GNPDA1*, *GOT1*, *HADHB*, *HPRT1*, *MGST1*, *POLD2*, *POLE3*, and *SHMT2*). In addition, the Lasso- Cox regression derived gene correlation coefficient was used to analyze the risk score which led to the classification of the samples as either high-risk or low-risk. The patients with high risk had significantly lower survival rates than those with low risk as evidenced by Kaplan Meier analysis.

Additionally, the risk scores’ sensitivity and accuracy in predicting oral cancer prognosis at one, three, and five years were established using the pROC package ROC function. Based on the AUC values obtained from ROC analysis, the model involving 11 MRGs was found to have the potential to serve as a prognostic instrument with good predictive sensitivity and accuracy.

Moreover, results from univariate and multivariate Cox regression analyses on risk scores and clinical characteristics (age, gender, stage, grade, T stage, and N stage) demonstrated that the risk score could independently predict the prognosis. Further, clinical correlation analysis on risk scores’ and clinicopathological features confirmed the significant relationship between MRGs-derived risk scores and clinical features inclusive of stage, grade, and sex.

Later, the outcome of the training model was verified using the validation set and was found to be similar for all the parameters analyzed reinstating the accuracy of the prognosis forecasting capability of the model. Following this, a nomogram was constructed considering age, gender, TNM stage, and risk score, adopting the R software RMS package to predict the survival status. The results revealed that the nomogram well predicted the survival status of oral cancer patients with accuracy as compared to other indicators such as gender, grade, T stage, N stage, and risk score.

Further, to understand the gene function, enrichment analysis was performed using GO and KEGG databases. The resultant gene set enrichment data was analyzed by GSEA (29). GO analysis revealed that MRGs were predominantly linked with the glycosyl compound metabolic process, nucleoside metabolic process, purine ribonucleoside monophosphate metabolic process, and purine nucleoside monophosphate metabolic process, while KEGG analysis showed that MRGs were mostly enriched in carbon metabolism, drug metabolism-other enzymes, phenylalanine metabolism, and purine metabolism amongst others. GSEA (29) showed that cysteine and methionine metabolism, β-alanine metabolism, pyrimidine metabolism, and purine metabolism were highly associated with high-risk groups, whereas α-linolenic acid metabolism was enriched in low-risk patients.

In the next step, the association between immune cells and risk signature demonstrated that dendritic cells, immature dendritic cells, T helper cell 17, and mast cells were highly enriched in the low-risk group, whereas T helper cell 2 was more enriched in the high-risk group. This result confirms that low-risk patients have an active immune function and better prognosis than high-risk patients.

**Figure 8:**
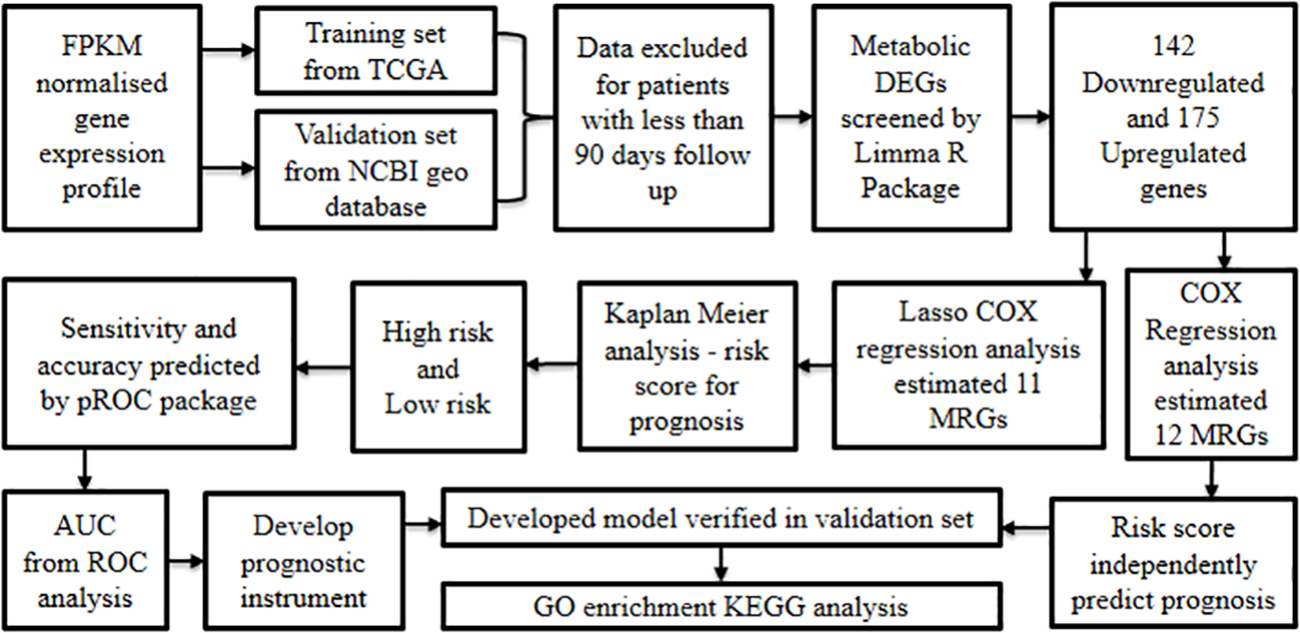
Development of oral cancer prognostic model based on metabolism-related genes.

Additionally, ScRNA-Seq data of GSE172577 was processed by the Seurat package followed by Principal Component Analysis to identify the main cell clusters. The findings showed that 31,719 single cells were grouped into seven major cell types: B cell, endotheliocyte cell, epithelial cells, fibroblasts, mast cell, myeloid cell, and T cells using marker genes. On further exploration of the expression, in the validation set, among the prognosis-related MRGs in various cells of oral cancer, it was found that five genes were predominantly expressed in epithelial cells and may be restricted to tumor cells, while the six other MRGs were expressed neither in tumor microenvironment nor in tumor cells (**Figure 8**). Conclusively, this MRGs-based model has the potential to predict the survival status of oral cancer patients and may assist in clinical decision-making for personalizing treatment.

### 4.4 Omics to predict metastasis - exploiting protein profiles to forecast oral cancer metastasis at the lymph node level

Yu et al., 2019 (63) developed a novel prediction model to forecast lymph node metastasis and assess the prognostic features in terms of survival rate in patients with early-stage oral cancer. This retrospective cohort study collated clinicopathological factors and potential biomarkers to construct the proposed model. This study was parsed into two stages: 1) the training stage and 2) the validation stage.

At the outset, a total of 159 oral cancer patients were recruited in the study, wherein, 58 patients (33 without lymph node metastasis and 25 with lymph node metastasis) were assigned to the training set and the rest of the patients were allocated to the validation set (74 without lymph node metastasis and 27 with lymph node metastasis).

Subsequently, the oral cancer tissues or oral cancer metastasis cell lines from the above cases were subjected to mass spectrometry to capture the proteins that (a) expressed different protein abundances between the metastatic and non-metastatic oral cancer conditions, (b) exhibited significant association with clinical features, (c) displayed positive staining with oral cancer tissues compiled in The Human Protein Atlas (58), and (d) were highlighted in oral cancer metastasis-related literature. This in-depth analysis revealed eight candidate biomarkers such as prolyl 4- hydroxylase, alpha polypeptide II (P4HA2), caldesmon (CAD), bone marrow stromal cell antigen 2 (BST2), integrin beta-6 (ITGB6), protein-glutamine gamma-glutamyl transferase 2 (TGM2), peroxiredoxin-4 (PRDX4), superoxide dismutase (SOD2), and thrombospondin-1(THBS1) of relevance in oral cancer metastasis. Further, the expression levels of the aforesaid protein biomarkers were analyzed by performing immunohistochemistry. The resultant Histochemistry score (H-score) revealed a significant elevation in the expression levels of CAD, BST2, PRDX4, ITGB6, and SOD2 in oral cancer lymph node metastatic tissues compared to non-metastatic tissues. On the other hand, the H-Score indicated that P4HA2, TGM2, and THBS1 did not display a significant differential expression between the tissues. The proteins with significantly elevated expression captured by H-score were later subjected to binary logistic regression analysis. This signified a substantial association of SOD2 and CAD with lymph node metastasis evinced via the histopathological grade status and their expression levels. Further, univariate and multivariate Cox regression analysis performed on SOD2 and CAD demonstrated a significant correlation of the survival status with histopathological grade status, SOD2 expression, CAD expression, and lymph node metastasis. A prediction model for life status constructed based on the Kaplan Meier analysis correlated poor histopathological grade status, high expression levels of SOD2 and CAD, and positive nodal metastasis with poor OS and DFS.

The predictions of the training model were corroborated by leveraging the patient data reserved for the validation stage. In accordance with the prediction model, based on immunohistochemistry and regression analysis, significantly higher expression levels of SOD2 and CAD alongside poor histopathological grade status were linked with oral cancer nodal metastatic characteristics. Similarly, Kaplan Meier analysis of the validation set confirmed the association of high expression levels of SOD2 and CAD with poor OS and DFS. The constructed model based on protein profile serves as a sensitive tool in capturing the metastatic potential at an earlier stage in order to achieve a desirable prognostic value.

## 5 Discussion

The rapidly evolving bioinformatics and ML tools have unfurled the applications of omics to comprehend the underlying mechanisms of a disease on a molecular basis (64). The associated clinical intricacies are addressed by untangling the molecular anomalies at the epigenomic, genomic, transcriptomic, proteomic, and metabolomic levels (65). Disorders such as oral cancer, bearing strong genetic underpinnings especially derive benefits from an amalgamation of the above strategies (66).

This review presents the applicability of omics strategies in unraveling the key molecular mechanisms to confront the clinical complexities pertinent to diagnosis, management, and prognosis encountered in oral cancer (67). The diagnosis section provides a landscape of methodologies to address the constraints regarding early diagnosis, noninvasive biomarker identification, differential diagnosis, tumor characterization, and classification. Biomarker discovery is a crucial strategy to target these setbacks, particularly to enable early diagnosis. However, a significant concern with regard to the high dimensionality of enormous omics data lies in selecting informative genes with substantial biological relevance to clinical outcomes and to translate this big data into meaningful patterns with key genes and pathways. This problem is addressed in a study by Wan et al., 2021 (7), which emphasizes the significance of studying aberrant DNA methylation patterns and its effects on gene expression and metabolic pathways in order to find biomarkers for oral cancer. In this study, an integrated multi-omics strategy was employed to explore DEGs and MeDEGs with potential clinical applicability.

Biomarkers and their expression patterns can also be selected as features to build diagnostic models using ML and optimize the process. On this note, Yang et al., 2020 (16) explored the TCGA database to select DEmRNAs and DElncRNAs of oral cancer and further utilized ML to screen key lncRNAs with diagnostic and prognostic potential. Based on the findings, an interaction network was constructed and functional annotation was performed in the co-expressed DEmRNAs of lncRNAs. By combining the gene expression profile and clinical data, the study identified clinically relevant biomarkers, establishing a biological basis for further research into oral cancer.

Furthermore, salivaomics analysis which utilizes saliva as a source of biochemical data is a lucrative approach that can overcome the drawbacks associated with solid biopsy sampling such as invasiveness, difficulty in sample collection, and storage (68). The study by Adeoye et al., 2022 (18) presents a comprehensive framework for carrying out the salivary methylome biomarker-based oral cancer diagnosis using genome-wide techniques with ML assistance. Tumor characterization is an essential step in categorizing cancer, and it can aid in identifying molecular signatures that can support early detection, tumor staging, differential diagnosis, and individualized treatment (69). The work by Shafana et al., 2021 (20) provides an approach to classify oral cancer into five molecular subclasses that could potentially assist in early diagnosis of oral cancer as well as individualize drug therapy. Since the co-expression networks demonstrated that the majority of the genes are differentially expressed at an early stage rather than a later one, this approach is very promising particularly in the context of early diagnosis. Additionally, omics is explored through bioinformatics techniques to capture the carcinogenic propensity of other oral disorders sharing similarities with oral cancer. In this regard, de Lanna et al., 2022 (26) studied the commonalities in the gene signatures of OLP and oral cancer to elucidate the malignant potential of the former and identified potential therapeutic targets for the same.

In the prognosis section, an emphasis is placed on reconnoitering biomarkers underlying the progression of the disease and collating them to develop models that predict survival outcomes. By using a bioinformatics approach, Reyimu et al., 2021 (56) performed prognostic studies in oral cancer to identify prognostic hub genes with good reliability and sensitivity. This study gives scope for future research to confirm these hub genes in prospective clinical trials and understand the role of these indicators in contributing to oral carcinogenesis. Furthermore, prognostic models developed to predict metastasis at an early stage can be utilized to derive clinically pre-emptive decisions and initiate prompt intervention (70). Early-stage prognostic prediction is especially significant as it can aid in selecting the most suitable treatment for patients with oral cancer, thereby improving their survival. In order to address oral cancer metastasis and treatment related concerns, it is the need of the hour to mine prognosis-related molecular markers that reflect the biological characteristics of the tumor. Numerous databases, such as TCGA, GEPIA, GEO, KEGG, and DAVID among others, have made extensive gene expression data and associated clinical data available to researchers. Decoding these databases using various bioinformatics strategies can uncover pathways to arrive at treatment plans, develop new prognostic models with high precision from the standpoint of tumor cell biological behavior (62).

Finally, with respect to oral cancer management, the area of interest majorly lies in utilizing omics strategies to identify novel therapeutic targets and carry out target-guided drug discovery (71). Drug repurposing is one of the breakthrough concepts used to discern new therapeutic options for oral cancer. This concept is delineated in the study by Siam et al., 2021 (41), where they evaluated the efficacy of two CYP inhibitors to be repurposed as an Akt pathway inhibitor in the treatment of oral cancer. The study generated a precise and definite view of the efficiency of the repurposable drugs by examining their interactions with related disease pathways using diverse *in silico* methodologies. Another challenging aspect of oral cancer therapy is drug resistance and associated therapeutic failure, the review also sheds some light on the application of omics strategies to overcome the same (72). On these lines, proteomic and phosphoproteomic data were combined in the study by George et al., 2022 (49) to identify kinases with druggable potential in chemotherapy-resistant tongue cancer.

Further, in advanced stages of oral cancer, surgical intervention becomes the mainstay of clinical management. This review highlighted an intriguing application of omics in recognizing biomarkers that complement surgery in terms of demarcating the surgical margins from normal tissues to assist precision surgery techniques (73). In conclusion, the review has exemplified the essential role of various technology-driven omics strategies to reconnoiter solutions to clinical constraints related to oral cancer diagnosis, management, and prognosis.

## Author contributions

KsS, KaS, and GS: these authors contributed equally to this work and share first authorship. KsS and GS have contributed in conceptualization and literature selection. KaS, KsS, GS, and SC have contributed in manuscript writing and development. KaS, PD, and KN have contributed in developing schematics in addition to manuscript writing. DA, SV, SK, VH, IH, FA, KA, and SP have reviewed and revised the manuscript. All authors contributed to the article and approved the submitted version.

## Glossary

**Table:**

ADMET	Absorption, Distribution, Metabolism, Elimination, Toxicity
AUC	Area Under Curve
BST2	Bone Marrow Stromal Cell Antigen 2
CAD	Caldesmon
DAVID	Database for Annotation, Visualization and Integrated Discovery
DCA	Decision Curve Analysis
DEG	Differentially Expressed Gene
DElncRNA	Differentially Expressed long non coding RNA
DEmRNA	Differentially Expressed mRNA
DFS	Disease Free Survival
DMC	Differentially Methylated CpG
DMG	Differentially Methylated Gene
DMR	Differentially Methylated Region
ECM	Extracellular Matrix
ExtraTrees	Extremely Randomized Trees
GEPIA	Gene Expression Profiling Interactive Analysis
GO	Gene Ontology
GSEA	Gene Set Enrichment Analysis
HNSCC	Head and Neck Squamous Cell Carcinoma
H	Score Histochemistry score
ITGB6	Integrin Beta 6
KEGG	Kyoto Encyclopedia of Genes and Genomes
LASSO	Least Absolute Shrinkage and Selection Operator
LC	MS/MS Liquid Chromatography-Tandem Mass Spectrometry
lncRNA	long non-coding RNA
MAPK1	Mitogen-activated protein kinase 1
MeDEG	Methylation-regulated and Differentially Expressed Gene
MRG	Metabolism-related Gene
NBI	Narrow Band Imaging
NCBI	GEO - National Center for Biotechnology Information - Gene Expression Omnibus
OLP	Oral Lichen Planus
OPMD	Oral Potentially Malignant Disorders
OS	Overall Survival
P4HA2	Alpha Polypeptide II
PPI	Protein-Protein Interaction
PRDX4	Peroxiredoxin-4
ROC	Receiver Operating Characteristic
RRBS	Reduced Representation Bisulfite Sequencing
SOD2	Superoxide Dismutase-2
STRING	Search Tool for the Retrieval of Interacting Genes
SVM	Support Vector Machine
TCGA	The Cancer Genome Atlas
TGM2	Protein-glutamine gamma-glutamyl transferase 2
THBS1	Thrombospondin-1
